# Similarities and differences between bone quality parameters, trabecular bone score and femur geometry

**DOI:** 10.1371/journal.pone.0260924

**Published:** 2022-01-11

**Authors:** Kyong Young Kim, Kyoung Min Kim

**Affiliations:** 1 Department of Internal Medicine, Gyeongsang National University Changwon Hospital, Changwon, South Korea; 2 Department of Internal Medicine, Seoul National University Bundang Hospital and Seoul National University College of Medicine, Seongnam, South Korea; 3 Division of Endocrinology, Department of Internal Medicine, Yongin Severance Hospital, Yonsei University College of Medicine, Yongin, Republic of Korea; University of Life Sciences in Lublin, POLAND

## Abstract

Bone quality is a critical factor that, along with bone quantity, determines bone strength. Image-based parameters are used for assessing bone quality non-invasively. The trabecular bone score (TBS) is used to assess quality of trabecular bone and femur geometry for cortical bone. Little is known about the associations between these two bone quality parameters and whether they show differences in the relationships with age and body mass index (BMI). We investigated the associations between the trabecular bone score (TBS) and femur cortical geometry. Areal bone mineral density (BMD) was assessed using dual energy X-ray absorptiometry (DXA) and the TBS was assessed using iNsight software and, femur geometry using APEX (Hologic). A total of 452 men and 517 women aged 50 years and older with no medical history of a condition affecting bone metabolism were included. Z-scores for TBS and cortical thickness were calculated using the age-specific mean and SD for each parameter. A ‘discrepancy group’ was defined as patients whose absolute Z-score difference between TBS and cortical thickness was > 1 point. TBS and cortical thickness correlated negatively with age both in men and women, but the associations were stronger in women. Regarding the associations with BMI, TBS provided significant negative correlation with BMI in the range of BMI > 25 kg/m^2^. By contrast, cortical thickness correlated positively with BMI for all BMI ranges. These bone quality-related parameters, TBS and cortical thickness, significantly correlated, but discordance between these two parameters was observed in about one-third of the men and women (32.7% and 33.4%, respectively). Conclusively, image-based bone quality parameters for trabecular and cortical bone exhibit both similarities and differences in terms of their associations with age and BMI. These different profiles in TBS and FN cortical thickness might results in different risk profiles for the vertebral fractures or hip fractures in a certain percentage of people.

## Introduction

Osteoporosis is a skeletal disorder characterized by low bone mass and deterioration of bone quality, which increase the vulnerability of the bone and the risk of fracture [1]. As the population ages, the incidence of osteoporosis and number of osteoporotic fractures are growing and becoming a major public health problem, which is related to higher morbidity, mortality, and medical costs [2]. In addition, problems including chronic disabling pain, gait disturbance, deformity, and performance impairment in daily life may occur after fracture and can reduce the quality of life, especially in the elderly population [3]. Therefore, early detection of people with a higher risk of fractures and timely appropriate intervention are important.

Bone strength is determined by the composite of bone mass and bone quality [4]. A quantitative parameter, bone mass is assessed by measuring bone mineral density (BMD) by dual-energy X-ray absorptiometry (DXA), and the severity of low BMD is diagnosed as osteopenia or osteoporosis using the T-score value [5]. The risk of fracture is higher in people with osteoporosis, although the frequency of fragility fractures is higher in those with osteopenia [6]. This means that bone quality, another parameter that determines bone strength in addition to bone mass, is also critical to the risk of fractures in some people.

Bone quality reflects several factors related to bone structural and material properties [7] and image-based noninvasive parameters, such as the trabecular bone score (TBS) and femur geometry has been used to assess bone quality. The TBS, that was proposed by Pothuaud et al. evaluates the pixel grey-level variations in DXA images of the lumbar spine and is used to assess the bone quality of trabecular bone [8]. Femur geometry represents the distributive property of hip bone mass and is closely related to the strength of hip cortical bone [9]. Among the femur geometric parameters, the cortical thickness in the femur neck is known to be most closely related to the femur strength [10]. Previous studies have shown that the TBS and femur geometry are independent predictors of fracture [6,11,12].

The femur and lumbar spine have different compositional properties: femur bone is composed by mainly cortical bone, and lumbar spine contains mainly trabecular bone [13,14]. The bone biology differs somewhat between cortical bone and trabecular bone, which means that the clinical factors that affect bone can also differently influence bone according to the bone site. Based on these, several studies have compared bone quality between femur and spine [15–17]. However, it has not been clearly elucidated yet whether bone quality-related parameters of the spine and femur might differ in terms of their associations with age and body mass index (BMI), the two most critical clinical factors affecting bone quality. The aims of our study were to investigate the effects of age and BMI on the TBS and geometry, and to determine whether these two parameters differ in certain proportion of people.

## Materials and methods

### Subjects and design

This is a single-center, cross-sectional study and we consecutively included men and peri- or postmenopausal women aged >50 years who had undergone DXA as screening for osteoporosis at Seoul National University Bundang Hospital (SNUBH, Seongnam, South Korea) from January 2015 to March 2016. Participants were excluded if they (i) were younger than 50 years old; (ii) were taking any medication (e.g., systemic steroids, hormone-replacement therapy, bisphosphonates, calcium or vitamin D replacement) and/or had any preexisting medical condition (e.g., parathyroid disease, hyper- or hypothyroidism, rheumatoid arthritis, or asthma) that could affect bone metabolism; (iii) had malignancy or chronic liver/renal disease; or (iv) had a previous major fracture. This study was approved by the Ethical Review Board of SNUBH (IRB No. B-1510/318-110) and informed consent from the patients or their legal representatives was waived. All the data were fully anonymized before we accessed them.

### Bone mineral density and hip structural analysis

BMD was measured in g/m^2^ at the site of the lumbar spine (L1–L4), femoral neck (FN), and total hip (TH) using DXA equipment (Discovery W; Hologic, Bedford, MA, USA) following the manufacturer’s protocol. Further analysis of geometric bone structure properties was performed using the hip structure analysis (HSA) program included in the APEX software (Hologic). The HSA program yields geometric data such as cross-sectional area (CSA, cm^2^), cross-sectional moment of inertia (CSMI, cm^4^), mean cortical thickness (mm), section modulus (cm^3^), and buckling ratio for each of the narrow neck (NN) regions [18]. Among those parameters, FN cortical thickness was used for the analyses as a representative parameter of femur geometry.

### Trabecular bone score

The TBS value was evaluated simultaneously with the lumbar spine in the DXA imaging using TBS iNsight software (version 2.1; Med-Imaps, Bordeaux, France). TBS value was determined as the mean value of the individual measurements for vertebrae L1-L4 [19].

### Statistical analysis

The baseline characteristics of the patients grouped by gender were compared using Student’s *t* test for continuous variables and Pearson chi-square test for categorical variables. The data are presented here as frequency (percentage) or mean (SD) as appropriate. The Shapiro-Wilk test was used to assess normality of BMI, TBS, and each parameter of femur geometry. Correlations of age or BMI, with TBS or femur geometry, and between TBS and femur geometry, were investigated using Pearson’s correlational analysis.

The Z scores for the TBS or FN cortical thickness were calculated using the mean and standard deviation (SD) values of the corresponding parameters in the same age group by the following equation: (actual TBS/ or FN cortical thickness value–mean TBS/ or FN cortical thickness value)/ SD of TBS/ or FN cortical thickness for the same age groups. To identify baseline characteristics associated with a discrepancy between the TBS and cortical thickness, we defined ‘discrepancy group’ as patients whose absolute Z-score difference between TBS and cortical thickness was > 1 point, and further categorized that group into two subgroups as follows. Patients with a TBS Z-score minus cortical thickness z-score >1 were categorized into the “TBS-dominant group.” Patients with a score less than –1 were categorized into the “cortical thickness-dominant group.” Patients with a score between 1 and –1 were categorized into the “no discrepancy group.” The baseline characteristics were compared between groups using ANOVA for continuous variables and the Pearson chi-square test for categorical variables. When a significant difference was detected by ANOVA, Bonferroni’s correction for three groups was used to identify the source of the difference. Statistical significance was set at *p* < 0.05 for two-tailed tests. All statistical analyses were performed using Stata/SE software (version 14.0; Stata Corp, College Station, TX, USA).

## Results

A total of 969 patients aged >50 years of age were included: 517 peri- or postmenopausal women and 452 men. Table 1 shows the clinical characteristics, the BMD, T-score, and bone quality parameters including TBS and femur geometry. All values showed normal distributions in Shapiro-Wilk test. The mean age was 64.7 ± 9.4 years in men and 66.7 ± 9.1 years in women. The mean BMI was 24.7 ± 3.1 kg/m^2^ in men and 25.0 ± 3.5 kg/m^2^ in women. The lumbar spine BMD and T-score were 1.035 ± 0.198 g/cm^2^ and 0.5 ± 1.5 in men, and 0.861 ± 0.171 g/cm^2^ and –1.1 ± 1.2 in women, respectively. The TBS was 1.353 ± 0.088 in men and 1.255 ± 0.097 in women. All values were significantly lower in women than in men (*p* < 0.001 for each comparison). All bone-related parameters, including TH BMD, FN BMD, and FN geometric parameters, were also significantly lower in women than in men. (*p* < 0.001 for each comparison).

**Table 1:** Baseline clinical characteristics and bone profiles of study subjects.

		Men (n = 452)	Women (n = 517)	*p-value*
Age (years)		64.73 ± 9.42	66.65 ± 9.10	*0*.*001*
Height (cm)		166.9 ± 7.1	153.3 ± 5.9	*<0*.*001*
Weight (kg)		69.1 ± 11.4	59.0 ± 9.1	*<0*.*001*
BMI (kg/m^**2**^)		24.71 ± 3.13	25.01 ± 3.50	*0*.*163*
BMI subgroups, n (%)				*0*.*758*
BMI ≤ 23 kg/m^2^		124 (27.43)	148 (28.63)	
23 < BMI ≤ 25 kg/m^2^		110 (24.34)	132 (25.53)	
BMI > 25 kg/m^2^		218 (48.23)	237 (45.84)	
Calcium (mg/dL)		8.93 ± 0.46	9.00 ± 0.44	*0*.*017*
Phosphorus (mg/dL)		3.49 ± 0.60	3.62 ± 0.54	*<0*.*001*
BUN (mg/dL)		16.12 ± 6.74	15.47 ± 5.32	*0*.*094*
Creatinine (mg/dL)		0.91 ± 0.73	0.75 ± 0.38	*<0*.*001*
eGFR		86.84 ± 25.44	91.14 ± 25.09	*<0*.*001*
***Lumbar Spine***				
Lumbar spine BMD (g/cm^2^)		1.035 ± 0.198	0.861 ± 0.171	*<0*.*001*
Lumbar spine LS T-score		0.544 ± 1.465	-1.149±1.237	*<0*.*001*
TBS L1-4 (unitless)		1.353 ± 0.088	1.255 ± 0.097	*<0*.*001*
***Hip***				
Total hip BMD (g/cm^2^)		0.902 ± 0.134	0.743 ± 0.124	*<0*.*001*
Total hip T score		-0.417 ± 1.025	-1.346 ± 1.243	*<0*.*001*
Femur neck BMD (g/cm^2^)		0.934 ± 0.170	0.787 ± 0.148	*<0*.*001*
Femur neck T-score		-0.805±1.412	-1.903±1.242	*<0*.*001*
Femur neck geometry				
	CSA (cm^2^)	3.217 ± 0.603	2.422 ± 0.422	*<0*.*001*
	CSMI (cm^4^)	3.410 ± 0.919	2.036 ± 0.502	*<0*.*001*
	Neck width (cm)	3.631 ± 0.297	3.258 ± 0.273	*<0*.*001*
	Cortical thickness (cm)	0.179 ± 0.035	0.151 ± 0.030	*<0*.*001*
	Section modulus (cm^3^)	1.708 ± 0.376	1.113 ± 0.228	*<0*.*001*
	Buckling ratio	11.648 ± 3.134	12.861 ± 3.772	*<0*.*001*

The data are expressed as mean ± SD. BMI, body mass index; TBS, trabecular bone score; BMD, bone mineral density; CSA, cross-sectional area; CSMI, cross-sectional moment of inertia; BUN, blood urea nitrogen; eGFR, estimated glomerular filtration rate.

### Association between age and TBS or FN cortical thickness

**Fig 1:**
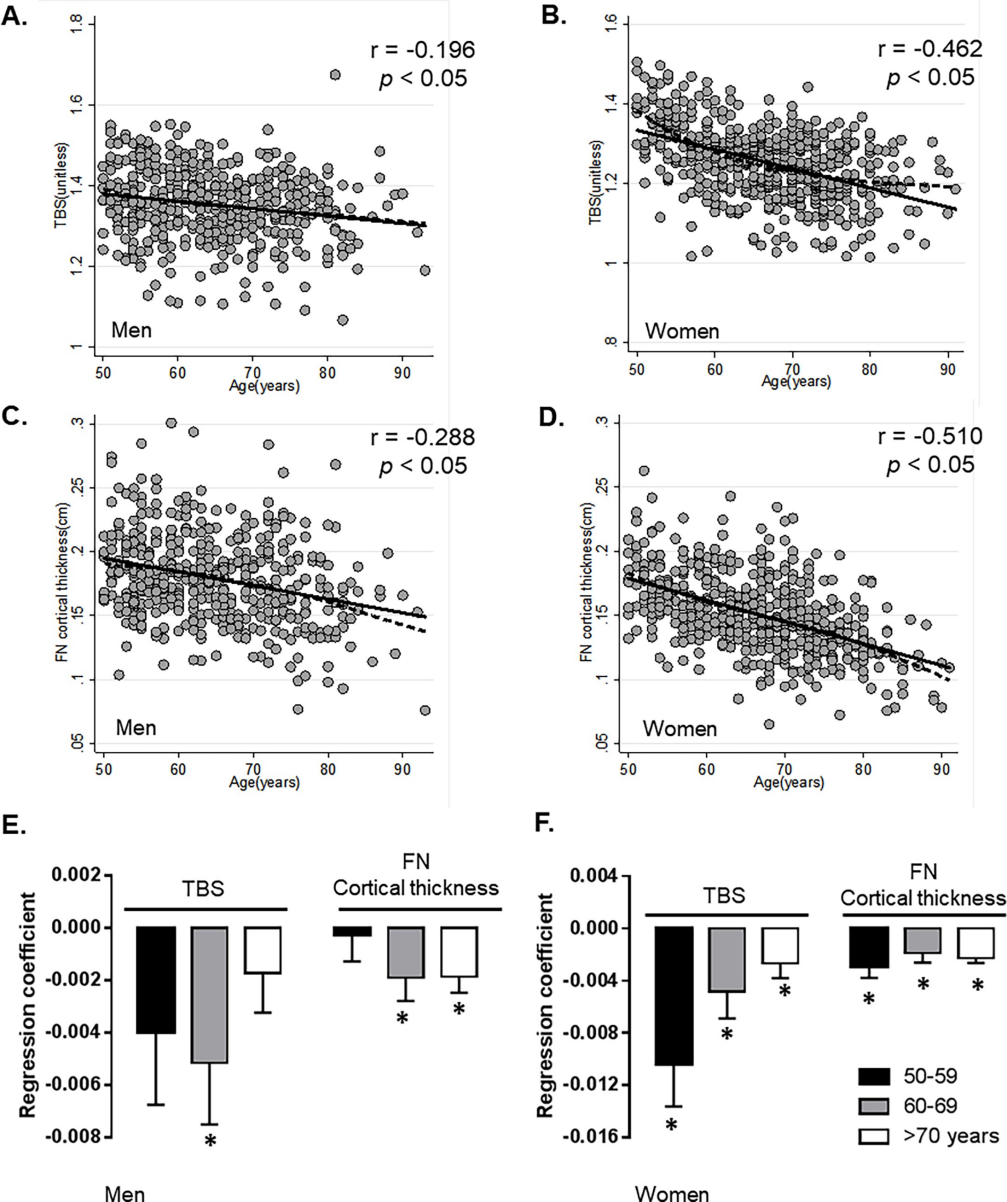
Correlation between age and TBS, or FN cortical thickness in men and women. (**A, B**). Simple linear regression (solid line) and lowess curve (dashed line) between age and TBS in men and women. r, Pearson’s coefficient. (**C,D**). Simple regression (solid line) and lowess curve (dashed line) between age and FN cortical thickness in men and women. r, Pearson’s coefficient. (**E,F**) Regression coefficient between age and TBS, or FN cortical thickness in each age group.* p<0.05. TBS, trabecular bone score; FN, femur neck.

Fig 1 shows the correlations between age and TBS and FN cortical thickness in men and women. Age correlated negatively with both TBS and FN cortical thickness in both men and women, although the association was stronger in women than in men (TBS, r = –0.196 for men, –0.462 for women; cortical thickness, r = –0.288 for men, –0.510 for women; *p* < 0.05 for each comparison) (Fig 1A–1D). Patients were grouped further into three categories according to age: 50–59, 60–69, and >70 years. The TBS correlated significantly with the age group 60–69 years, and FN cortical thickness correlated negatively with the age groups 60–69 years and >70 years in men (Fig 1E). In women, the strongest correlations were observed in the 50–59 years age group for TBS, and significant negative correlations across all age groups for FN cortical thickness (Fig 1F).

### Association between BMI and TBS or FN cortical thickness

**Fig 2:**
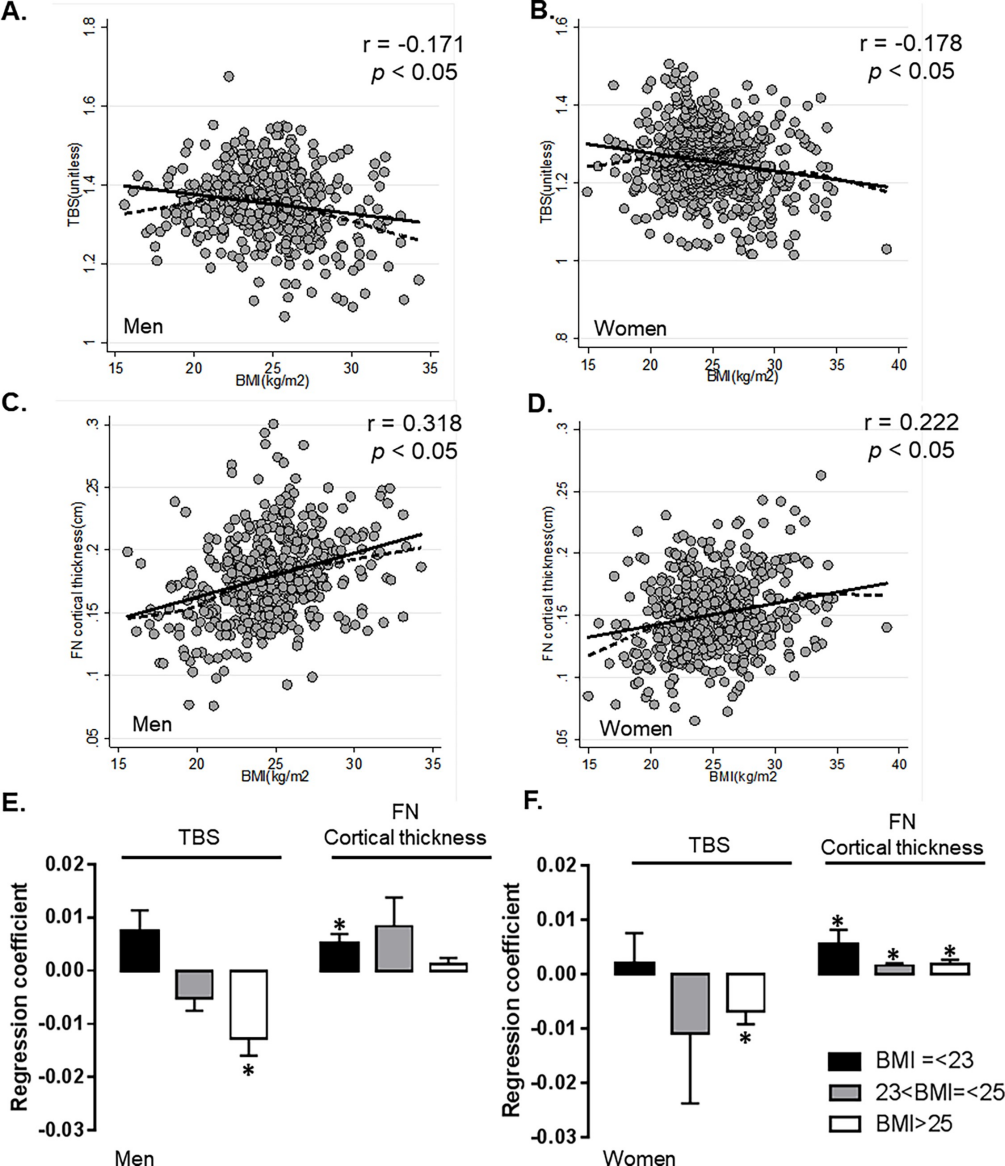
Correlation between BMI and TBS, or FN cortical thickness in men and women. (**A, B**). Simple linear regression (solid line) and lowess curve (dashed line) between BMI and TBS in men and women. r, Pearson’s coefficient. (**C,D**). Simple regression (solid line) and lowess curve (dashed line) between BMI and FN cortical thickness in men and women. r, Pearson’s coefficient. (**E,F**) Regression coefficient between BMI and TBS, or FN cortical thickness in each BMI group.* p<0.05. BMI, body mass index; TBS, trabecular bone score; FN femur neck. r, Pearson’s coefficient.

Fig 2 shows the associations between BMI and TBS, or FN cortical thickness. TBS provided generally negative correlations with BMI in both men and women (Fig 2A and 2B). In contrast, FN cortical thickness was correlated positively with BMI in both gender (Fig 2C and 2D). The patients were further stratified into three groups according to BMI: low or normal body weight (≤ 23kg/m^2^), overweight (> 23 to ≤ 25kg/m^2^), and obese (> 25kg/m^2^) according to the Asian population categories [20]. Analysis of the three BMI subgroups showed that the association between BMI and TBS differed between the non-obese and obese subgroups in both men and women. TBS did not correlate significantly with BMI <23kg/m^2^, but correlated negatively with BMI >25kg/m^2^ in both men and women (*p* < 0.05). By contrast, BMI correlated positively with FN cortical thickness across all BMI ranges (r = –0.318 for men vs. r = –0.222 for women, *p* < 0.05 respectively) (Fig 2).

### Discrepancy in z-score between TBS and FN cortical thickness

**Fig 3:**
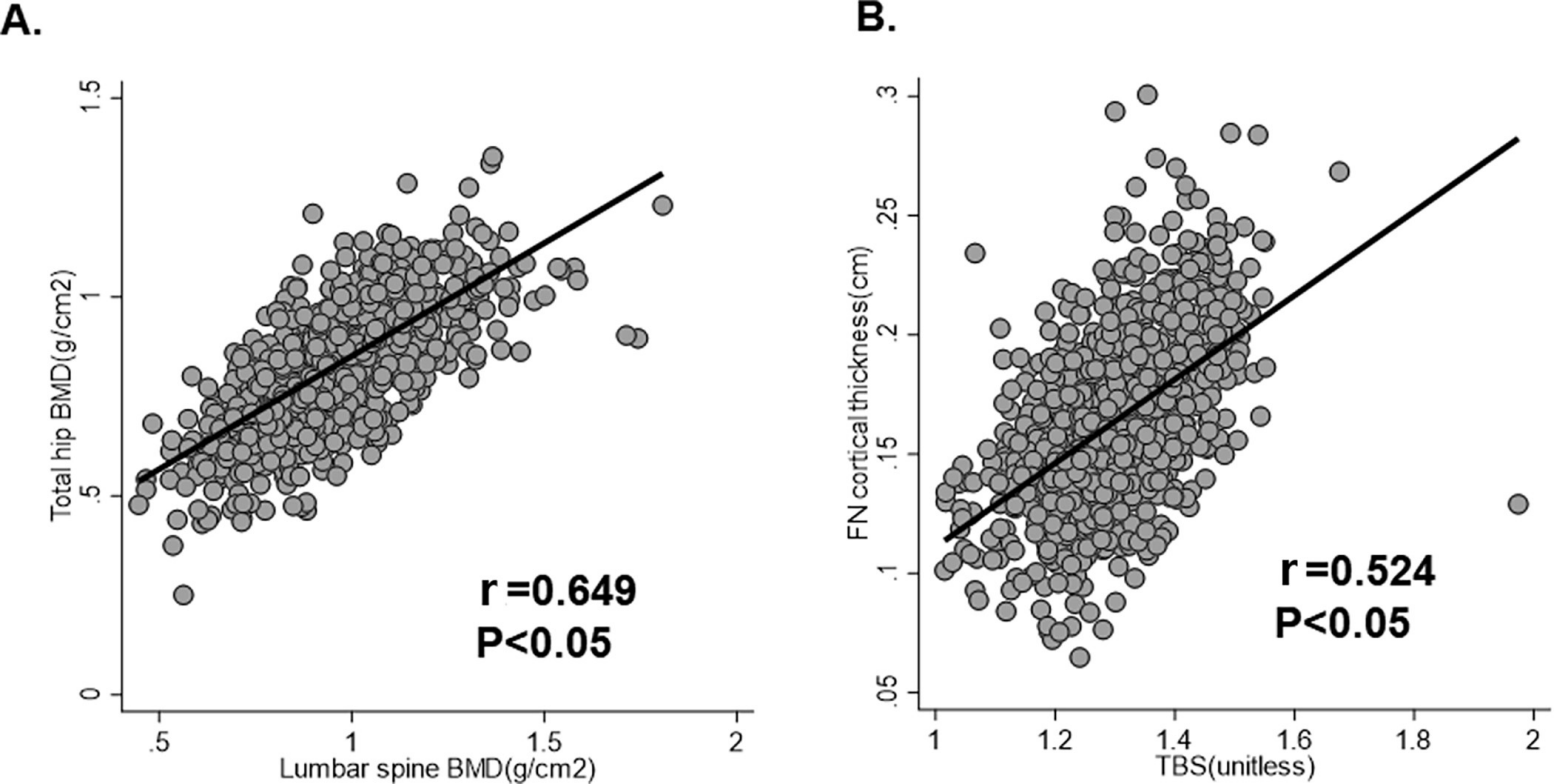
Correlation between total hip BMD and lumbar spine BMD, and between TBS and FN cortical thickness. TBS, trabecular bone score; FN, femur neck; r, Pearson’s coefficient.

Fig 3 displayed the association between lumbar spine BMD and total hip BMD, and the association between TBS and FN cortical thickness: The lumbar spine BMD correlated positively with TH BMD (Fig 3A) (r = 0.649, *p* < 0.05). The TBS and FN cortical thickness also correlated positively (r = 0.524, *p* < 0.05).

Despite of these strong positive correlations between BMDs at lumbar spine and total hip, and between TBS and FN cortical thickness, about one-third of patients had a discrepancy between z-scores for TBS and FN in both men and women (32.7% in men, and 33.3% in women) (Table 2). The “TBS-dominant group” had lower BMI than the “No discrepancy group”, whereas the “FN cortical thickness-dominant group” had a higher BMI in both men and women. Except for these bone-related parameters, the other parameters did not differ between the TBS-dominant and FN cortical thickness-dominant groups (Table 3).

**Table 2:** Discrepancy between TBS and FN cortical thickness.

	Male, n (%)	Female, n (%)	Total, n (%)
No discrepancy	304 (67.3)	345 (66.7)	649 (67.0)
TBS_Z-score_-FNCT_Z-score_> 1	74 (16.4)	84 (16.3)	158 (16.3)
FNCT_Z-score_-TBS_Z-score_> 1	74 (16.4)	88 (17.0)	162 (16.7)

TBS, trabecular bone score; FN, femur neck; FNCT, femur neck cortical thickness.

**Table 3:** Differences in clinical characteristics and bone parameters of discrepancy group in men and women.

**A. Men**				
	No discrepancy	Discrepancy group		
		TBS dominant	FNCT dominant	*P-value*
		TBS_Z-score_-FNCT_Z-score_> 1	FNCT_Z-score_-TBS_Z-score_> 1	
Age (years)	64.6 (9.313)	66.3 (10.93)	63.9 (8.086)	*0*.*25*
Height (cm)	166.6 (6.77)	166.4 (6.354)	167.7 (6.134)	*0*.*374*
Weight (kg)	68.3 (9.708)^a^	63.1 (10.04)^b^	76.8 (9.146)^c^	***<0*.*001***
BMI (kg/m^2^)	24.6 (2.889)^a^	22.7 (2.683)^b^	27.3 (2.819)^c^	***<0*.*001***
Calcium (mg/dL)	8.9 (0.447)	9.0 (0.565)	8.9 (0.39)	*0*.*508*
Phosphorus (mg/dL)	3.5 (0.602)^a^	3.4 (0.606)^a^	3.4 (0.566)^a^	***0*.*047***
BUN (mg/dL)	16.2 (7.492)	15.8 (4.737)	15.9 (4.994)	*0*.*856*
Creatinine (mg/dL)	0.93 (0.876)	0.87 (0.248)	0.84 (0.189)	*0*.*545*
LS BMD (g/cm^2^)	1.036 (0.188)	1.006 (0.232)	1.060 (0.204)	*0*.*788*
LS T-score	0.6 (1.459)	0.3 (1.315)	0.8 (1.599)	*0*.*058*
LS TBS	1.359 (0.074)^a^	1.423 (0.077)^b^	1.264 (0.088)^c^	***<0*.*001***
FN BMD (g/cm^2^)	0.939 (0.143)^a^	0.791 (0.162)^b^	1.056 (0.182)^c^	***<0*.*001***
FN T-score	-0.8 (1.207)^a^	-1.9 (1.351)^b^	0.2 (1.531)^c^	***<0*.*001***
FN cortical thickness	0.180 (0.029)^a^	0.150 (0.032)^b^	0.204 (0.037)^c^	***<0*.*001***
**B. Women**				
	No discrepancy	Discrepancy group		
		TBS dominant	FNCT dominant	P-value*
		TBS_Z score_-FNCT_Z score_> 1	FNCT_Z score_-TBS_Z score_> 1	
Age (years)	66.5 (8.726)	67.8 (10.904)	66.1 (8.678)	*0*.*413*
Height (cm)	153.9 (5.859)	152.4 (6.648)	153.1 (5.339)	*0*.*096*
Weight (kg)	59.5 (8.452)^a^	53.1 (8.542)^b^	62.9 (10.197)^c^	***<0*.*001***
BMI (kg/m^2^)	25.1 (3.21)^a^	22.8 (3.044)^b^	26.8 (3.895)^c^	***<0*.*001***
Calcium (mg/dL)	9.0 (0.415)	9.0 (0.521)	9.1 (0.424)	*0*.*187*
Phosphorus (mg/dL)	3.6 (0.541)	3.5 (0.57)	3.6 (0.514)	*0*.*386*
BUN (mg/dL)	15.1 (5.072)	16.3 (5.543)	16.1 (5.963)	*0*.*092*
Creatinine (mg/dL)	0.75 (0.42)	0.79 (0.336)	0.74 (0.184)	*0*.*603*
LS BMD (g/cm^2^)	0.862 (0.140)	0.850 (0.290)	0.867 (0.128)	*0*.*251*
LS T-score	-1.1 (1.237)	-1.4 (1.322)	-1.1 (1.132)	*0*.*122*
LS TBS	1.257 (0.084)^a^	1.324 (0.102)^b^	1.178 (0.088)^c^	***<0*.*001***
FN BMD (g/cm^2^)	0.791 (0.128)^a^	0.656 (0.122)^b^	0.897 (0.149)^c^	***<0*.*001***
FN T-score	-1.9 (1.081)^a^	-2.9 (1.133)^b^	-1.1 (1.265)^c^	***<0*.*001***
FN cortical thickness	0.151 (0.026)^a^	0.124 (0.024)^b^	0.173 (0.031)^c^	***<0*.*001***

All data are shown as mean (standard deviation).

*Analyses were done with ANOVA with Bonferroni correction and a, b, c values not sharing the same superscript are significantly different at a p<0.05.

BMI, body mass index; BUN, blood urea nitrogen; LS, lumbar spine: BMD, bone mineral density; TBS, trabecular bone score; FN, femur neck; FNCT, femur neck cortical thickness.

## Discussion

In this study, we investigated the relationships of TBS and FN cortical thickness between age and BMI, critical determinants of bone strength in treatment-naïve patients. We found that both TBS and FN cortical thickness correlated negatively with age, as predicted. However, some differences were observed; the TBS declined rapidly with aging, and it was more pronounced in relatively younger age group (<70 years old in men and, <60 years old in women). In contrast, FN cortical thickness provided modest decline with aging, and started to decline only after 60 years old in men and decreased similarly in all age groups in women after their 50s. There was also a significant difference in the association with BMI. The TBS provided significant negative correlations with a BMI in only obese subjects with BMI>25kg/m^2^ in both men and women. However, FN cortical thickness correlated positively for all BMI categories from normal to overweight.

BMD is a major determinant of bone strength and is the most reliable measure for assessing fracture risk. However, BMD has limited accuracy for assessing bone strength in certain conditions, such as the presence of vertebral compression fractures, aortic calcification, or osteophytes, all of which increase with age [21,22]. Moreover, in other pathological conditions including lytic bone disease, chronic inflammatory disease, laminectomy, metallic materials, or obesity, BMD has limited ability in predicting fracture risk [22]. For these reasons, BMD by itself may not be the best measure for assessing bone strength. Many osteoporotic fractures occur in people with osteopenic BMD levels [6].

Bone quality is another critical component of bone strength, and bone turnover rate, mineralization, accumulation of microdamage, and bone geometry are bone quality-related properties. Despite the clinical importance of bone quality in determining bone strength, most of these parameters require invasive assessment and therefore have limited clinical application. However, several image-based indirect methods have been proposed for evaluating bone quality, and the TBS and FN cortical thickness are now used widely in clinical practice [9,11,12]. Several studies have shown that these parameters are associated with vertebral and non-vertebral osteoporotic fractures in postmenopausal women and men. The TBS is lower in patients with a prior osteoporotic fracture than in those without a fracture history, regardless of the presence of osteoporosis or osteopenia [6,23,24]. Other studies have shown that femur geometry is associated with increased risk of hip fracture [12,25,26].

There was a general trend that BMD shows similar tendency in site-specific measurement, but several studies have reported a discrepancy in BMD between the lumbar spine and hip [16,27]. That is, some people with osteoporosis exhibit a discrepancy in BMD T-score between their lumbar spine and hip, which may mean that the risk of vertebral and hip fractures may differ according to the individual patient’s pattern of BMD in different sites [16,28]. The early postmenopausal state and secondary osteoporosis (caused by hyperthyroidism, malabsorption, liver disease, rheumatoid arthritis, or medication) can lead to decreased lumbar spine BMD. By contrast, compression fracture, osteophytosis, and aortic calcification increase lumbar spine BMD. Vitamin D deficiency affects the hip rather than the spine. In this study, we observed significant associations between age and BMI, the main determinants of bone mass and strength, and the TBS and FN cortical thickness. The TBS reflects earlier bone loss, and FN cortical thickness seems to show later bone loss compared to other in both genders. This may reflect differences in the bone properties at each site. Bone loss begins earlier and proceeds faster in trabecular bone (typical of the lumbar spine) than in cortical bone (typical of the femur) because of higher bone-resorbing site in trabecular structures [29,30].

There is a more-pronounced difference in the association between BMI and the two bone quality parameters: TBS and FN cortical thickness. Lumbar spine BMD correlates positively with BMI, but BMD can be overestimated in obese people because of their central obesity [31]. However, in contrast to the relationship between BMD and BMI, one study reported a negative association between obesity and the TBS [32], although another study reported no association between BMI and TBS [33]. In the present study, TBS correlated negatively only in the group with BMI>25kg/m^2^, but not in the group with BMI<25 kg/m^2^. Obesity increases bone marrow adiposity, and greater fat mass and bone marrow adiposity may contribute to the lower TBS [34].

In contrast to the TBS, FN cortical thickness correlated positively with BMI in all BMI categories, and the strongest positive association was in the group with a BMI<23 kg/m^2^. This positive association was weakened or disappeared in the overweight and obese groups. Obesity imposes a greater mechanical loading on bone, which causes the bone mass to increase as it adapts to the greater load. This loading mechanism could be a protective mechanism, especially in the hip area [35,36].

Our study also showed a discrepancy between TBS and FN cortical thickness in about one-third of patient. The TBS-dominant group had a normal BMI, but the FN cortical thickness-dominant group had a higher BMI compared with the no discrepancy group. This finding suggests that the risk of future vertebral or hip fracture may differ according to the region-specific bone quality status, which means that some patients may be at higher risk of vertebral fracture but not hip fracture, or *vice versa*. Therefore, physicians should assess patients individually according to their fracture risk as indicated by their region-specific bone quality parameters.

There are several limitations of this study. This study was a hospital-based study, and the results may not be generalizable to the general population. Bone-related parameters such as parathyroid hormone or vitamin D levels and bone turnover markers were not included in the present study. Furthermore, FN cortical thickness was assessed by DXA that was not originally designed for assessing geometric parameters. Although strong positive correlations have already been reported between DXA- and QCT-derived geometric parameters in the previous studies [37], further studies using different imaging modalities to evaluate bone geometric parameters were needed to confirm these observations. In addition, history of fractures and maternal fractures could not be investigated because of the retrospective study design. A further limitation is that few people with very high or very low BMI were included. Despite these limitations, our study has some advantages. This is the first study to analyze the relationship between femur geometry and TBS according to age and BMI in large number of treatment-naïve patients. This study provides clinical information that may be useful for predicting femur and lumbar strength, and hip and vertebral fracture risk on an individual basis.

## Conclusions

Image-based bone quality parameters of trabecular and cortical bone have both similarities and dissimilarities in terms of their associations with age and BMI. The TBS value showed drastic and earlier changes with age, whereas modest and constant changes were observed in FN cortical thickness. A higher BMI is associated with a greater FN cortical thickness, indicating greater femoral strength, but negatively affects TBS values and may be detrimental to vertebral strength in the range above 25 kg/m2. These different profiles of TBS and FN cortical thickness with age and BMI could result in the different risk profiles for the vertebral fractures or hip fractures in given individuals and a considerable percentage of adults aged>50 years may have different bone strength profiles depending on the skeletal sites.

## Supporting information

S1 Data (XLS)
